# A Global Genome Segmentation Method for Exploration of Epigenetic Patterns

**DOI:** 10.1371/journal.pone.0046811

**Published:** 2012-10-12

**Authors:** Lydia Steiner, Lydia Hopp, Henry Wirth, Jörg Galle, Hans Binder, Sonja J. Prohaska, Thimo Rohlf

**Affiliations:** 1 Junior Professorship for Computational EvoDevo, Institute of Computer Science, University of Leipzig, Leipzig, Germany; 2 Interdisciplinary Center for Bioinformatics, University of Leipzig, Leipzig, Germany; 3 Leipzig Research Center for Civilization Diseases, University of Leipzig, Leipzig, Germany; 4 Max-Planck-Institute for Mathematics in the Sciences, Leipzig, Germany; National Institutes of Health, United States of America

## Abstract

Current genome-wide ChIP-seq experiments on different epigenetic marks aim at unraveling the interplay between their regulation mechanisms. Published evaluation tools, however, allow testing for predefined hypotheses only. Here, we present a novel method for annotation-independent exploration of epigenetic data and their inter-correlation with other genome-wide features. Our method is based on a combinatorial genome segmentation solely using information on combinations of epigenetic marks. It does not require prior knowledge about the data (e.g. gene positions), but allows integrating the data in a straightforward manner. Thereby, it combines compression, clustering and visualization of the data in a single tool. Our method provides intuitive maps of epigenetic patterns across multiple levels of organization, e.g. of the co-occurrence of different epigenetic marks in different cell types. Thus, it facilitates the formulation of new hypotheses on the principles of epigenetic regulation. We apply our method to histone modification data on trimethylation of histone H3 at lysine 4, 9 and 27 in multi-potent and lineage-primed mouse cells, analyzing their combinatorial modification pattern as well as differentiation-related changes of single modifications. We demonstrate that our method is capable of reproducing recent findings of gene centered approaches, e.g. correlations between CpG-density and the analyzed histone modifications. Moreover, combining the clustered epigenetic data with information on the expression status of associated genes we classify differences in epigenetic status of e.g. house-keeping genes versus differentiation-related genes. Visualizing the distribution of modification states on the chromosomes, we discover strong patterns for chromosome X. For example, exclusively H3K9me3 marked segments are enriched, while poised and active states are rare. Hence, our method also provides new insights into chromosome-specific epigenetic patterns, opening up new questions how “epigenetic computation” is distributed over the genome in space and time.

## Introduction

Genome-wide measurement and analysis of transcript levels have led to a different understanding of transcriptional regulation in mammalian cells (ENCODE) [1], [2]. It has become obvious that the genome is pervasively transcribed and that chromatin structure impacts transcription and the resulting transcripts levels in various ways. In order to understand these regulatory effects of chromatin, new assays for studying genome-wide chromatin modification have been introduced [3], [4].

Part of the regulatory effects is ascribed to histone modifications. All types of histones, namely H2A, H2B, H3, and H4, can be modified at multiple sites, i.e. specific amino acid residues. During modification, chemical groups, such as acetyl and methyl groups, biotin, small proteins, or sugars become attached to target sites. In the following, we will consider a specific modification at a specific residue of one of the histones as an epigenetic mark.

The function of epigenetic marks can be versatile. It is known that trimethylation at histone H3 lysine 4 (H3K4me3) marks euchromatin and positively correlates with transcription [5]–[8]. In contrary, trimethylation at histone H3 lysine 27 (H3K27me3) is involved in formation of heterochromatin, and transcriptional silencing [8], [9]. Although the effects of H3K4me3 and H3K27me3 seem conflicting, they can be found together at the promoters of genes for cell differentiation in ESCs [10]. Genes in bivalently marked chromatin are in a poised state and can be activated by removing the H3K27me3 or stably repressed by removing the H3K4me3 mark [11], [12]. Likewise H3K27me3, trimethylation at histone H3 lysine 9 (H3K9me3) is mainly linked to repression of transcription and repressive DNA methylation [13]. It has been shown that the gene transcriptional activity depends on the combination of histone modification marks and sequence specific features. In particular, histone modification pattern of H3K4me3, H3K27me3 and H3K9me3 have been demonstrated to differ at promoters with high and low CpG-density [11], [14]. This is associated with gene function as housekeeping genes are frequently associated with CpG-rich promoters while low levels of CpG-density are rather found at promoters of tissues-specific genes.

In [15], [16], the histone code hypothesis suggests that combinations of epigenetic marks provide a regulatory output that differs from the sum of the regulatory effects of individual marks. Furthermore, cross-talk between epigenetic marks has been suggested given examples such as avoidance of H3K9me2 and phosphorylation of H3S10 [17]. Thus, comparative analysis of multiple modifications promises new insights into chromatin regulation, its interplay with gene expression and chromatin structure formation.

Chromatin immunoprecipitation combined with next generation sequencing (ChIP-seq [18]) enables fast measurement of the genome-wide distribution of various epigenetic marks in multiple cell states. However, most of the existing analysis strategies do not enable a comprehensive visualization of concerted interrelations within such data sets.

Most of the existing approaches visualize epigenetic data on the basis of the underlying DNA sequence in a linear fashion, for example, as UCSC genome browser track. Individual gene loci, and the relative position of peaks in the sequencing read density and gene annotation are of focal interest [19], [20]. For this type of analysis, several software packages are available (EpiChIP [21], Repitools [22], chromatin based expression prediction [23]). These approaches enable powerful analysis of relationships between specific genes and associated epigenetic marks, they are naturally limited, however, with respect to the identification of global correlations. A thorough analysis of such higher order patterns requires additional preprocessing of the data, in particular a systematic partitioning (segmentation) with respect to genomic loci.

In the context of genome-wide gene expression data, similar problems arise which have been successfully addressed by a number of techniques, e.g. self-organizing maps (SOMs) [24], [25]. In the following, we present a method to analyze complex, high-dimensional epigenetic data by combining a versatile approach for combinatorial segmentation of modification data with SOMs. SOMs are an unsupervised clustering method and a convenient tool to reduce multi-dimensional data to low-dimension by condensing information and visualization as mosaic images. This representation is easy to interpret as the SOM organizes in an intuitive fashion. Thus, it facilitates data exploration without prior formulation of detailed hypotheses while visual clues can still be ascribed to the associated input data points. Furthermore, the method enables integration of auxiliary quantitative information, such as CpG-density and transcriptional activity in the analysis.

After introducing the technical details, we apply our method to H3K4me3, H3K27me3 and H3K9me3 histone modification data in mouse embryonic stem cells (ESC), mouse embryonic fibroblasts (MEF) and mouse neuronal progenitor cells (NPC) measured by Mikkelsen et al. [11]. The set of modifications is comprised of activating (H3K4me3) and repressive marks (H3K27me3, H3K9me3) that can coexist in the same region. It is known that these mark play a key role in differentiation. Besides this, H3K4me3 and H3K9me3 show antagonistic behavior with respect to correlation with DNA methylation. Thus, may provide insights into cross-regulation of epigenetic marks. We demonstrate that the method provides new insights even into such a well-studied set of data and discuss the broad spectrum of possible further applications.

## Results

In the following, we introduce i) a genome segmentation method based on multiple histone modification data in different cell types and ii) a method to compress the modification pattern of the resulting thousands of segments into two-dimensional images which allows a sample-to-sample comparison of the different modifications in the different cell types. In general, this requires data for 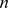 different modifications in 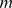 different cell types.

### Mapping and preprocessing

We downloaded ChIP-seq data on 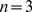 histone modifications (i.e. H3K4me3, H3K27me3 and H3K9me3) for 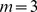 cell types, i.e. embryonic stem cells (ESCs), mouse embryonic fibroblasts (MEFs), and neuronal progenitor cells (NPCs) from GEO (accession number GSE12241) [11]. To ensure comparability, we reanalyzed the sequencing reads as follows.

Each ChIP-seq data set was mapped against the mm9 mouse genome download from UCSC genome browser using segemehl [26], an in-house tool for fast mapping of short sequences with insertions, deletions and mismatches. For further analysis we kept only those reads of length 26–36 nt that had at least 90% identity to a genomic locus. In addition to the modification ChIP-seq data, we also mapped the whole cell extract (WCE) sequencing data for all three cell types and the H3-ChIP-seq data available only for ESCs.

**Figure 1:**
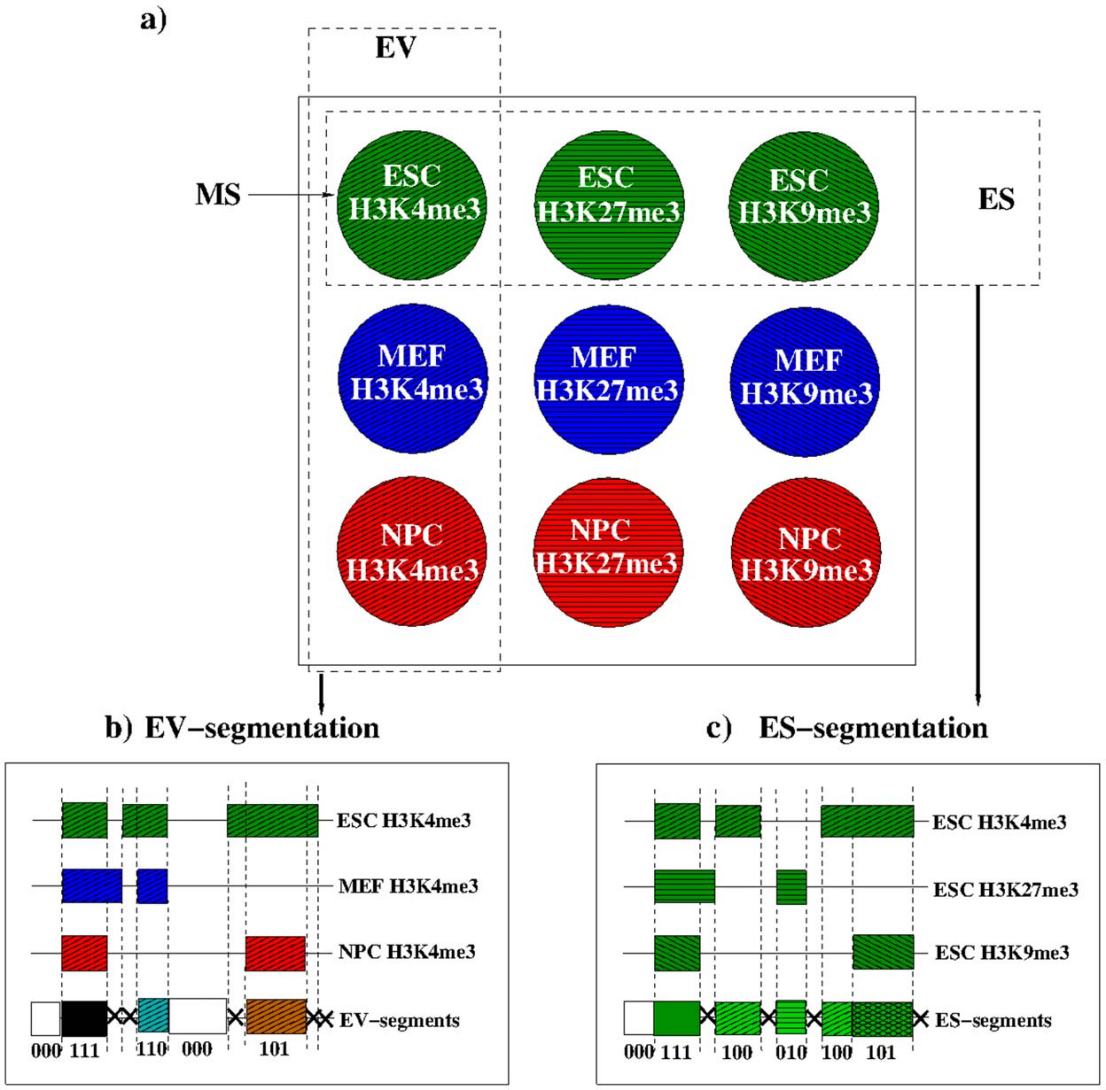
Segmentation of the whole genome. a) Given a set of modification states MS (patterns and colors encode modifications and cell types respectively), we performed either a segmentation based on epigenome states (ES-segmentation) or on epigenome variations (EV-segmentation). This is illustrated in b) and c). Horizontal lines represent the genome sequence and boxes illustrate the modified regions in each of the modification states (MSs) used. Vertical dashed lines represent the boundaries of the regions. Resulting segments are the regions between adjacent boundaries. Segments that are too small (indicated by X) are not kept. The combinatorial epigenetic profile (CEP) of each segment is characterized by a binary vector (e.g. (111) when all three modifications of reference are present).

For each data set, we counted the number of reads per genomic position. In ESCs, we validated the modification data with the H3 data and left reads for modified H3 aside when no reads from the H3 data set could be mapped to the same site. Doing this, less than 1% of positions was not validated which kept the ESC data comparable to the MEF and NPC data. We calculated read enrichment by dividing the positional read counts from modification data by the corresponding counts from the WCE data. Intensity data may provide important information, e.g. when it comes to quantifying impact of chromatin states on transcription and gene function in a detailed manner. We instead choose a binary representation of the data mainly for two reasons: 1) the amount of noise present in the ChIP-seq data reflected by extremely broad distributions of enrichment values, 2) the goal to visualize genome-wide reorganization of epigenetic patterns across different cell types, rather than applying a gene-centered (functional) view - this requires substantial reduction in the amount of input information. The discretization of the modification data is obtained by joining consecutive positions with a read enrichment of at least 3 into a modified region if their distance was smaller than 100 nt (less than one nucleosome). Resulting regions of length 99 nt or smaller were treated as unmodified to discard potentially erroneously mapped reads. The set of modified regions of one data set represents the modification state (MS). We can construct a MS for each of the 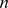 modifications and 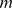 cell types, respectively. This yields a total of 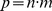 MSs (compare Figure 1a).

### Genome segmentation

In order to compress the data and to compare the distribution of the 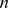 different modifications in the 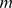 different cell types, we subdivide the genome into genomic segments. Each of them is characterized by the coverage value for each of the 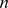 modification states in the 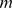 cell types. This results in a vector of dimension 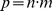 which we call epigenetic profile (EP) in the following.

In general, one could apply an arbitrary segmentation to the genome. A meaningful choice for segmentation should reflect the types of correlations one is interested in and allow for a reasonable level of data compression adequate for SOM-analysis. Hence, to emphasize either the variation among different marks from the same cell type or the variation among the same marks from different cell types, we examine the epigenome state (ES) or epigenome variation (EV), respectively, as illustrated in Figure 1.

As an example application for our method, we explain how to obtain the EPs for examination of the ES.

1. For a cell type of reference (here, ESC), all 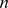 modification states are selected. Notice that the discretized representation of the chosen data holds a list of modified regions specified by the genomic position of the boundaries.
2. We then project all boundaries of modified regions from the 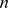 modification states onto the genome (ES-segmentation). The superposition of all boundaries subdivides the genome into segments. Short segments (here, length 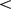
200 bp) are omitted in our applications since they are below the discretization limit (characteristic length of 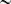
200 bp DNA per nucleosome).
3. As a consequence of segmentation, each segment is covered either 0% or 100% by each of the 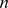 modification data sets used for segmentation (see Figure 1c). It is therefore possible to describe a segment 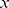 by an 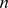-dimensional binary vector which we call combinatorial epigenetic profile (CEP), e.g. 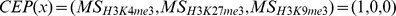. Evidently, we can observe at most 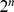 different combinatorial epigenetic profiles.
4. Information from the remaining 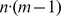, so far disregarded, modification states can be integrated. We therefore intersect the segment 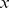 obtained in step 3 with the corresponding modified regions in the 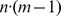 complementary data sets overlapping 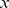. For each of these intersections 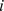, we calculate the amount of modification covering the segment 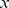. This leads to 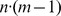 coverage values in the interval 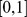. Appending these values to the CEP of 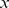, we finally obtain a 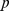-dimensional vector (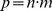), which we refer to as epigenetic profile (EP) of 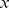.

To modulate the relative contribution of the coverage values to the EP we weight them using the factor 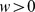 which is set to unity per default. Variation of this weighting factor allows us to modify the structure of the SOM images such that it is determined either more by the CEP- or more by the coverage-components of the EP (see below and File S1).

Analogously, an EV-segmentation can be performed by intersection of 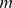 modification data for a modification of reference (here, H3K4me3) resulting in a 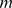-dimensional CEP, and a 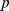-dimensional EP.

### Data Compression and Visualization by SOMs

For further analysis and visualization, we aim at sorting and compressing the segmentation data of dimension 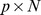 using self-organizing Maps (SOMs) [27]. Here, 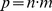 is the dimension of the epigenetic profiles and 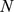 the number of segments obtained during segmentation. SOMs have been applied previously to molecular data, such as gene expression data and are reported to provide an intuitive and informative global representation of the data [25]. SOMs are trained using an unsupervised learning algorithm and are applied to high-dimensional data aiming at dimension reduction and a discretized representation of the input data (based on artificial neural networks). Thereby, the large number of input data points is projected onto a grid with a predefined geometry embedding 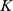 SOM-nodes. Specifically, we aim at projecting EPs to SOM-nodes. Accordingly, we assign to each node 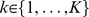 a 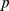-dimensional vector, further referred to as *meta-EP*. After an appropriate initialization, meta-EPs are adapted to EPs by a similarity-based learning procedure. In addition, inclusion of the local context ensures similarity of neighboring nodes in the SOM-topology.

In particular, we use a 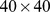 square grid (see File S2 for details) representing 1600 meta-EPs. We perform linear initialization of the meta-EPs to ensure deterministic SOM-procession [28]. Linear initialization extracts the two major properties (projections) structuring the data using principle component analysis. The first two principle components are mapped onto the width and height of the SOM, respectively. This ensures that the mean is located in the center of the SOM, while the strong effects are broken down along the sides. After initialization, the meta-EPs are updated during competitive learning in about 200.000 iterations over all EPs. Details are described in the Materials and Methods section. In this way, the EPs are placed on the map such that similar meta-EPs are located in close proximity while dissimilar meta-EPs are located more distantly. As the number of input segments 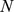 (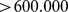) exceeds the number 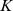 of nodes in the grid, each meta-EP represents a mini-cluster of, on average, about 400 segments. In our example, the actual, individual node occupancy ranges from 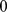 to 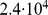 and 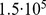 for the EV-segmentation and ES-segmentation, respectively.

The resulting SOM is finally visualized by mosaic images which we further refer to as SOM-images. Each of the tiles represents one node and its color corresponds to the respective meta-EP-value [29]. In addition to these SOM-images we generated population maps which visualize the number of EPs in each node as well as maps which visualize additional features such as the mean segment length. The construction of the supporting maps is described in the Materials and Methods section.

### Examples

We applied our segmentation and visualization method to the data set of Mikkelsen et al. after mapping and preprocessing as described in the Methods section. In a first example, we used ES-segmentation to study ***combinatorics of epigenetic marks in ESCs*** and integrated the modification data from the remaining cell types. In a second example, we applied EV-segmentation to study ***epigenetic variation among cell types with H3K4me3 as reference*** and integrated the modification data from the remaining epigenetic marks. We aim at highlighting characteristic features of our method and the benefit of the genome-wide exploration enabled.

#### ES-segmentation

**Figure 2:**
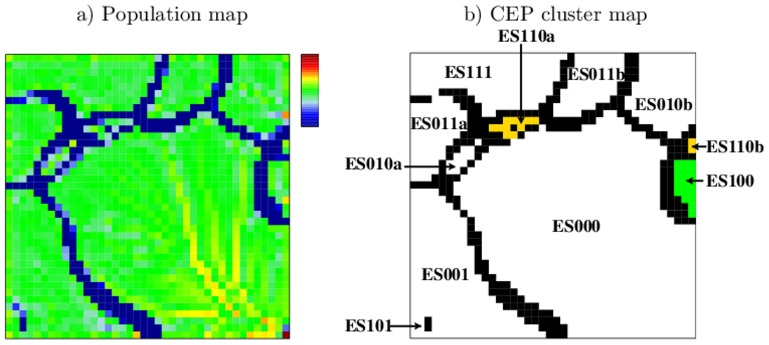
SOM-properties after ES-segmentation. a) The population map shows the number of EPs per node. It is logarithmically scaled and ranges from 0 (blue) to 150.000 (red). b) CEP cluster map. Islands with more than 10 EPs per node are shown in white and are labeled with their CEP. Borderlines with less than 10 EPs per node are shown in black. EPs with the same CEP cluster together in one island, except for ES110, ES010 and ES011 which split into two separated islands each (postfixes a,b). Colored are islands of active chromatin (ES100, green) and poised chromatin (ES110a,b, yellow).

ES-segmentation was applied to segment the whole genome with respect to H3K4me3, H3K27me3 and H3K9me3 modified regions in ESCs. The obtained set of EPs was subjected to SOM-learning as described above. The population map in Figure 2a shows the number of EPs assigned to each of the 1600 SOM-nodes after SOM-training. One can easily identify a certain number of green islands with a density of at least 10 EPs per node that are separated from each other by sparsely populated nodes with less than 10 EPs per node (dark blue).

We found that all EPs of each particular island refer to the same CEP. Figure 2b assigns the CEPs to the respective subregions of the map. All eight possible CEPs are present and allocated to eleven subregions of the SOM. Three CEPs, namely ES110, ES010, and ES011 locate to two different subregions of the SOM each, indicated by the postfixes “a” and “b”.

Hence, the structure of the SOM image and particularly its division into island-like regions is mainly governed by the CEP-part of the EP. We varied the value of the weighting factor between 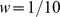 and 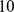 and trained new SOMs to assess its impact on the resulting SOM structure (see File S1). Decreasing weights at 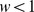 progressively reduce the SOM-textures to the underlying CEP-structure and partly merge the split “a” and “b” CEP subregions. In turn, CEP-islands not split for 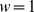 tend to divide into “a” and “b” subregions with increasing weight of the coverage part of the EP (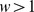). This trend is accompanied by the progressive smearing of the low-populated borders separating the different CEP-islands which partly disappear at large 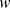. Adjacent CEP-islands tend to merge forming this way common regions of high (red) and low (blue) modification degrees. We choose an intermediate value of the weighting factor 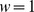 because it provides well resolved CEP-islands on one hand but also a moderate number of red high-modification spots. This choice allows relating a particular modification pattern to its reference state given by the respective CEPs and also to extract main trends of modification changes in the systems studied. It might be advisable to use 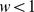 in future applications with a larger number of coverage components of the EP (e.g. if one considers a larger number of cell systems upon ES-segmentation and/or a larger number of histone modifications upon EV-segmentation) to obtain similar SOM-structures as discussed here.

The largest island of the SOM refers to the CEP ES000 representing the unmodified state in ESCs. It corresponds to 97% of the total chromatin. The CEP ES100 marks active chromatin in ESCs, i.e. only H3K4me3 modified (see green island in Figure 2b), ES110 indicates poised chromatin, which is H3K4me3 and H3K27me3 modified but not H3K9me3 modified (yellow islands in Figure 2b). All other CEPs mark different repressive states of chromatin.

**Figure 3:**
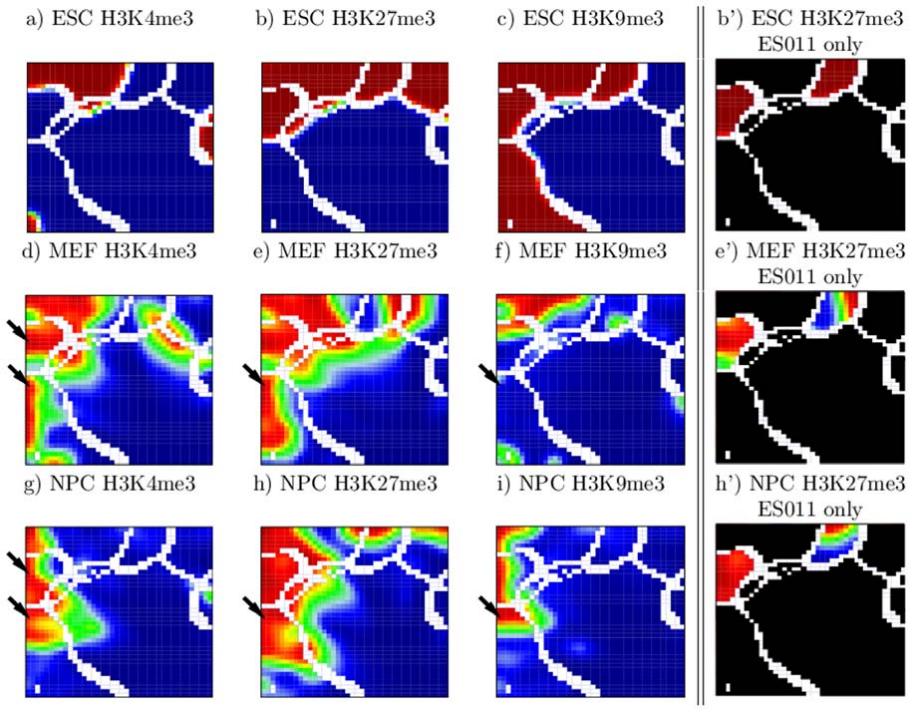
SOM-atlas after ES-segmentation. a)–i) Each tile in the mosaic images is colored by the average coverage of the segments assigned to it by the respective modification. High and low coverage is depicted in red and blue, respectively (linear scale). White tiles indicate the borderlines between islands with different CEPs (see also Figure 2). *De novo* formation of H3K4me3 and H3K27me3 in MEFs is a frequent event (black arrows in d and e). Many of these marks remain stable in NPCs (arrows in g and h). The arrow in panel f indicates poised chromatin *de novo* formed in MEFs. It partially turns into repressed chromatin in NPCs by H3K9me3 modification (arrow in i). b′), e′), and h′) Subregions corresponding to the same CEP, as ES011a and ES011b, differ by their H3K27me3 status in MEF and NPC.

Figure 3 shows the complete atlas of SOM-images after ES-segmentation, three images for each modification and each cell type, respectively. Here, the border lines of the CEP clusters are shown in white. The three SOM-images for ESCs provide the distribution of the CEPs ES1**, ES*1* and ES**1 in dark red, i.e. the distribution of segments modified at least by either H3K4me3, H3K27me3 or H3K9me3, respectively (compare with Figure 2b). For MEFs and NPCs, the EPs adopt continuous values due to the particular coverage of predefined segments with the respective modification. This causes the color gradient in Figure 3d–i.

Figure 3 demonstrates a genome-wide reorganization of the modification patterns during specification of ESCs into MEFs and NPCs. This reorganization includes de-modification of segments (red areas turn to blue ones) as well as *de novo* modification (blue areas turn to red ones). These changes appear to follow different rules for the different marks.

*De novo* trimethylation of H3K4 and/or H3K27 is observed during differentiation of ESCs into MEFs (see arrows in Figure 3d and 3e indicating regions which turn from blue in ESCs into red in MEFs). Many of these marks found in MEFs but not in ESCs are also observed in NPCs (see arrows in Figure 3g and 3h). Interestingly, most of the associated segments are in a poised chromatin state in MEFs (H3K4me3 and H3K27me3 modified but not H3K9me3 modified) but in an inactive state in NPCs (additionally modified with H3K9me3, arrows in Figure 3f and 3i). In contrast to H3K4me3 and H3K27me3, for which the amount of modified segments is roughly constant in all three cell types, a large-scale H3K9 de-methylation is observed in MEFs and NPCs compared to ESCs (all ES**1 islands turn, at least partly, into blue). *De novo* H3K9 tri-methylation of segments without H3K9me3 in ESCs (ES**0) is only rarely observed. Therefore, it turns out that H3K9me3 remodeling is almost exclusively restricted to segments carrying this mark in ESCs. Interestingly, the unmodified chromatin in ESCs (ES000) largely remains unmodified (colored in blue) also in MEFs and NPCs. Thus, while about 97% of the total chromatin is located in this region, changes in the chromatin state with respect to H3K4me3, H3K27me3 and H3K9me3 is mainly observed in the remaining 3% of the chromatin.

We found that SOM-training splits part of the islands referring to a particular CEP into two subregions distinguished with the postfixes “a” and “b” (see Figure 2b). Remarkably, all these paired subregions (ES110a/b, ES010a/b, and ES011a/b) strongly differ in their H3K27me3 modification status in MEFs and NPCs (see Figure 3e′ and 3h′). We labeled subregions with a high H3K27me3 coverage in MEFs and NPCs with a and those with a low coverage with b. The finding that segments with the same CEP in ESCs can acquire different modification states in MEFs compared to NPCs opens up interesting questions about the underlying mechanisms. Examples are possibly different modes of recruitment of the marks in ESCs or targeted (de-)modification events during differentiation into MEFs and NPCs. Further, these reorganization processes may depend on other histone marks not included in the current study.

**Figure 4:**
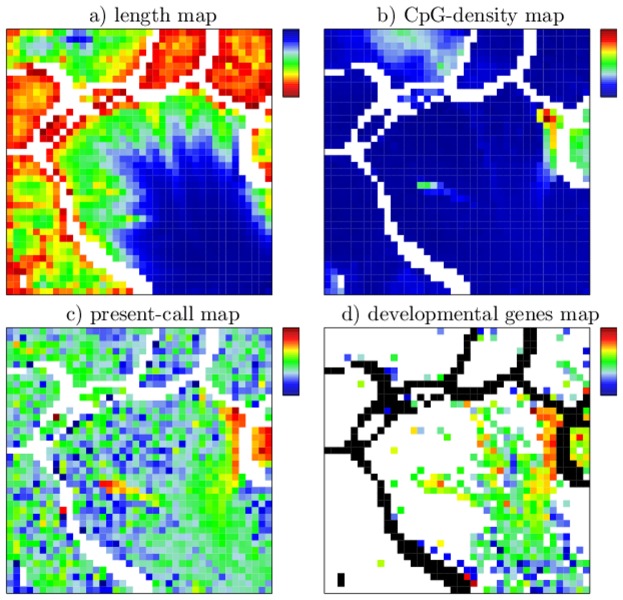
Supporting maps after ES-segmentation. a) Average segment length per node ranging from 200 (red) to 135.000 bp (blue, linear color scale). b) Average CpG-density of the segments per node ranging from 0 (blue) to 0.08 CpGs/bp (red, linear color scale). c) Fraction of segments that exclusively overlap with genes, which are significantly expressed (present) in all three cell types (low-to-high refers to blue-to-red). d) Fraction of segments overlapping with genes associated with the GO-term –cellular developmental process. A logarithmically scale is used ranging from 0.004 (dark blue) to 0.3 (red). Tiles of nodes without any overlap are colored in white.

Recruitment of modifications and its regulatory consequences can be further analyzed using complementary information summarized in supporting maps. Figure 4 shows supporting maps displaying the average segment length (a), the average CpG-density of a segment (b), the expression status of genes overlapping with a segment (c) and the distribution of segments overlapping developmental genes (d). Note that none of these additional data were used for SOM-training. Instead, they were projected onto the SOM-topology which is governed solely by the EPs (see Materials and Methods section).

In the segment length map (Figure 4a) one observes that long chromatin segments mostly accumulate in the island referring to segments unmodified in ESC (ES000 in Figure 4a). However, a small region of long segments is also observed in the island referring to triple-modified segments in ESCs (ES111). It has been demonstrated that the appearance of long modified segments may refer to cooperative mechanisms in recruitment of modifications [30], [31]. Accordingly, the appearance of long modified segments in triple-modified ESCs suggests cooperative recruitment of H3K4me3, H3K27me3 and H3K9me3 to chromatin.

Inspection of the CpG-density map (Figure 4b) reveals that segments carrying H3K4me3 in ESCs (ES1**) can be CpG-enriched. This has already been observed by Mikkelsen et al. [11] and is consistent with the finding that H3K4me3-modifying complexes include a binding motif for unmethylated CpGs [32]. However, beside in (ES1**) segments, CpG-enrichment is found for a subset of the unmodified segments (ES000) only. In particular, it is not found for segments that become *de novo* modified with H3K4me3 in MEFs and NPCs as e.g. part of the segments of (ES011). Hence, H3K4me3 recruitment in MEFs and NPCs is obviously not associated with high CpG-densities which suggests a different mechanism compared to the CpG-dependent recruitment in ESCs discussed above. Note that this alternative mechanism may exist also in ESCs, since ES111 and ES101 in part also contain segments with no CpG-enrichment. Correlations between H3K4me3 and CpG-density will be discussed later in the context of EV-segmentation.

H3K27me3 recruitment has been associated with high CpG-density as well [11]. Moreover, a H3K4me3 dependent mechanism has been suggested [33]. Indeed, in all cell types a large fraction of segments with a high H3K27me3 coverage lies in regions of the SOM that overlap regions of high H3K4me3 coverage. However, this overlap is not exhaustive. This is most obvious for segments of ES010 and ES011 in ESCs (see Figure 3a and 3b). Again, this observation opens up interesting research questions: it may either indicate that H3K4me3 is not necessarily required for H3K27me3 recruitment, or that previously existing H3K4me3 marks were removed at an earlier stage of development. Moreover, all ES01* islands lack CpG-enrichment. Consequently, H3K27me3 recruitment is also not necessarily associated with local CpG-enrichment. As the ES01* segments are predominately short segments (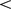
1 kb), and thus cooperative binding of modifying complexes is limited, we suggest H3K27me3 recruitment to ES01* to be sequence specific but not CpG-dependent. Different binding motifs for segments of ES01*a and ES01*b, would explain the observed differences in their modification status in MEFs and NPCs. However, in the case of ES110, the difference in the CpG-density (ES110a low, ES110b high) may contribute to the different modification status in MEFs and NPCs.

Note that the occurence of e.g. short ES01* segments could result from insufficient saturation of H3K27me3 ChIP-seq libraries. However, if this was the dominant effect, we would not expect to measure a large number of long H3K27me3 segments which are also H3K4me3 modified (ES11*). Instead we would expect that H3K4me3 modified segments are randomly associated with H3K27me3 modified segments. It is one of the advantages of our combinatorial method that it is quite robust against this kind of measurement biases in the data for single modifications.

The chromatin-associated information considered so far can be easily correlated with the expression status of genes. In Figure 4c and 4d, this is demonstrated for two important classes, namely housekeeping and developmental genes. We find that ESC chromatin associated with particularly high CpG-density, is either active (ES100) or can be at least viewed as not actively silenced (spots in ES000). Strikingly, as seen in Figure 4c, it associates with “active genes”, i.e. genes that are expressed in all three cell types (see Material and Methods section for details).

According to Mikkelsen et al., genes associated with high CpG-density and monovalent H3K4me3 in ESCs may be considered as “housekeeping genes”. Actually, compared to all other islands in the SOM, we found that ES100 shows a more than 10-fold enrichment in segments that have a housekeeping probability 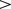
0.75 according to De Ferrari et al. [34]. By contrast genes associated with high CpG-density and bivalent chromatin (H3K4me3 and H3K27me3 modified) in ESCs have been linked to genes with more complex expression patterns among them key developmental genes [11]. Indeed, we see that segments of ES110b are associated with developmental genes (see Figure 4d). Strong enrichment, however, is mostly found in the CpG-rich spots of ES000.

In summary, ES-segmentation provides clear insights into genome-wide changes of epigenetic patterns during cell lineage specification and differentiation. Thus, it may point at extensive chromatin remodeling, since specific epigenetic patterns are associated with specific chromatin structures such as euchromatin and heterochromatin. Changes of the modification status of segments with a particular CEP in the reference system (here ESCs) can be easily detected and be used to formulate hypotheses e.g. on recruitment mechanisms. Supporting maps can be generated in order to evaluate or further detail these hypotheses. Moreover, chromatin structure can be linked straight forward to gene expression and function.

#### EV-segmentation

**Figure 5:**
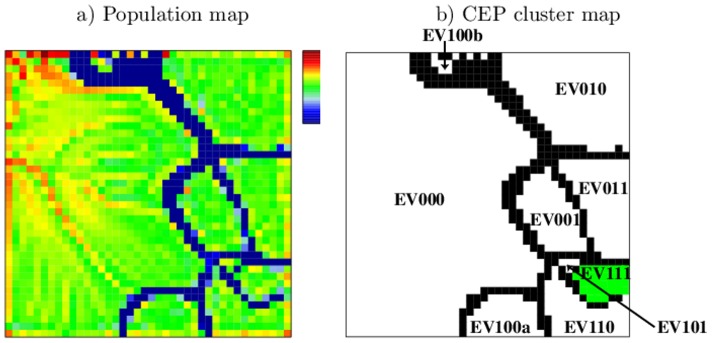
SOM-images after EV-segmentation. a) Population map. The number of EPs per node is logarithmically scaled and ranges from 1 (blue) to 24.000(red). b) CEP cluster map. Islands with more than 10 EPs per node are shown in white and are labeled with their CEP. Borderlines with less than 10 EPs per node are shown in black. Chromatin stable marked by H3K4me3 refers to EV111 (green island).

EV-segmentation was applied to segment the whole genome with respect to the variation among ESCs, MEFs and NPCs in the distribution of H3K4me3 modified regions. The so-obtained EP data set was subjected to SOM-training. The structures of the obtained population map and CEP cluster map are analogous to the corresponding maps after ES-segmentation (compare Figure 5 to Figure 2). The map shows highly populated islands that are separated by sparsely populated border zones. Again we found that all EPs of a particular island refer to the same CEP (see Figure 5b for assignments) The eight possible CEPs form nine islands where EV100 splits into two subregions labeled with “a” and “b”.

The largest part of the SOM is again occupied by unmodified segments, i.e. segments not carrying H3K4me3 modifications in any of the three studied cell types (EV000). EV000 corresponds to 98% of the total chromatin. Regarding the modified part of the chromatin, the CEP EV111 marks stable H3K4me3 modifications, while all other CEPs mark dynamic H3K4me3 modifications, i.e. segments that either lose or acquire this epigenetic mark in the course of differentiation.

**Figure 6:**
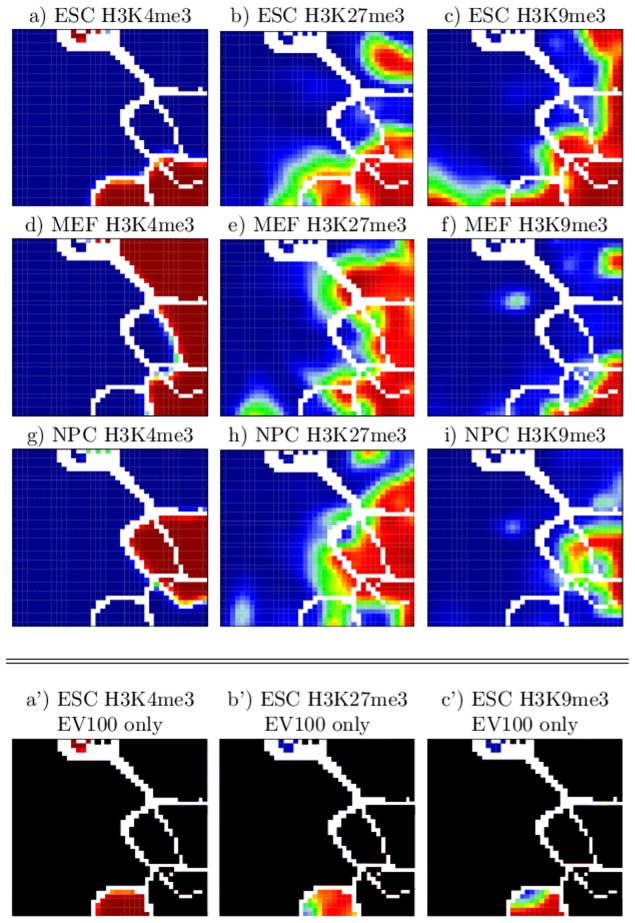
SOM-atlas after EV-segmentation. Color codes as in Figure 3. a′)–c′)Segments with CEP (EV100) locate to two islands EV100a and EV100b differing in their coverage by H3K27me3 (and H3K9me3) in ESCs.

Figure 6 shows the complete SOM-atlas obtained after EV-segmentation. It enables direct insights into the global reorganization of H3K4me3 modified chromatin during differentiation of ESCs into MEFs and NPCs. Inspection of the atlas shows that the paired subregions (EV100a/b) carrying H3K4me3 marks exclusively in ESCs (but not in MEFs and NPCs) can be distinguished by their combined H3K27me3 and H3K9me3 modification status in ESCs and thus by their chromatin activity status in these cells (see Figure 6a′–6c′). Quantification of the qualitative information demonstrate that 11065 out of 21759 (51%) segments carry repressive marks of which 6501 (48%) are in a repressed and only 685 (3%) in a poised chromatin state. An active chromatin state (H3K4me3 only) is observed in 4652 (21%) segments. While the “b”-subregion (EV100b) contains segments with activating marks only, EV100a is associated with segments of poised and repressive chromatin states, i.e. carrying also H3K27me3 and/or H3K9m3 marks. Having a closer look at the sub-regions in the island EV100a that show loss of H3K27me3 or H3K9me3 in either MEFs or NPCs, we conclude that H3K4me3 de-methylation is independent of repressive marks.

**Figure 7:**
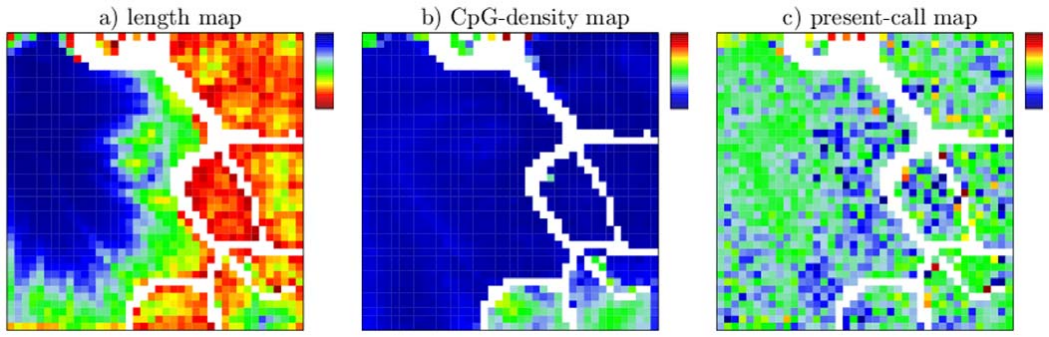
Supporting maps after EV-segmentation. a) Average segment length per node ranging from 200 (red) to 31.000 bp (blue). b) Average CpG-density of the segments per node ranging from 0 (blue) to 0.06 CpGs/bp (red). c) Fraction of segments that exclusively overlap with genes, which are significantly expressed (present) in all three cell types.

The supporting maps of the EV-segmentation (Figure 7) project additional information about the segment lengths, their CpG-density and the expression status of genes onto the SOM-topology. Long segments mostly accumulate in the EV000 island (see Figure 7a), i.e. they remain H3K4me3 unmodified under all conditions studied. A small amount of long segments associates with the EV110-island (see Figure 7a). These segments refer to H3K4me3 modified chromatin in ESCs that loose H3K4me3 in NPCs but not in MEFs.Segments of comparable length and modification status locate to island EV111 in the ES-segmentation maps. Consistently, the long segments of EV110 are modified with H3K27me3 and H3K9me3 in ESCs. Inspection of Figure 7b clearly shows that the associated DNA is enriched in CpGs. This suggest that the recruitment of the respective histone marks may thus be governed by the binding of modifying complexes to CpG-enriched chromatin as previously proposed [11], [32]. The association between the length of the segments, their CpG-density and histone modification status was already established using ES-segmentation, however, the orthogonal view via EV-segmentation more clearly shows that long and CpG-rich subregions lose H3K27me3 in NPCs when H3K4me3 is lost while the shorter and CpG-poor subregions more often stay H3K27me3 trimethylated.

Applying ES-segmentation we observed that many segments carrying the H3K4me3 mark in ESCs are enriched in CpGs. EV-segmentation clearly shows that such enrichment is present for EV100, EV100a and EV100b but only for a few spots of islands EV101 and EV111. The length (Figure 7a) and CpG-density maps (Figure 7b) clearly show that the EV111 segments are mostly short and CpG-poor. Interestingly, EV111 is the only large island with H3K4me3 marked segments in ESCs that are CpG-poor. In addition, EV111 is stably H3K4 methylated in all cell types. This surprising observation leads us to the conclusion that stable maintenance of H3K4me3 is CpG-independent. Quantitative analysis of the epigenetic state of the segments reveals that 10719 segments carry H3K4me3 in all cell types of which 6589 are either H3K9me3 or H3K27me3 marked in all cell types. Only 644 segments (about 9%) have no repressive marks either in ESCs, MEFs, or NPCs. Consistently, the present-call map (Figure 7c) indicates that EV111 segments do not associate with a higher fraction of active genes then other segments although being stably H3K4me3 modified in all three cell types.

The combination of supporting maps on segment length and CpG-density also shed light on the potential dependencies of H3K4me3 de-modification (EV100a,b). While the two islands for the CEP EV100 show no commonalities with respect to H3K27me3 and H3K9me3 marks, the do have short segment length and high CpG-densities in common. As a consequence, we conclude that only short, CpG-rich segments are H3K4me3 de-modified in MEFs (EV100a,b) while in NPCs also long, CpG-rich segments lose their H3K4me3 mark.

In summary, EV-segmentation enables more detailed insights into remodeling processes of the chosen reference modification (here H3K4me3) compared to ES-segmentation. However, as functional states appear to depend on the combinatorics of different modifications, EV-segmentation is expected to provide less information on the link between chromatin structure and gene function.

#### Chromosomal distribution of epigenetic marks

**Figure 8:**
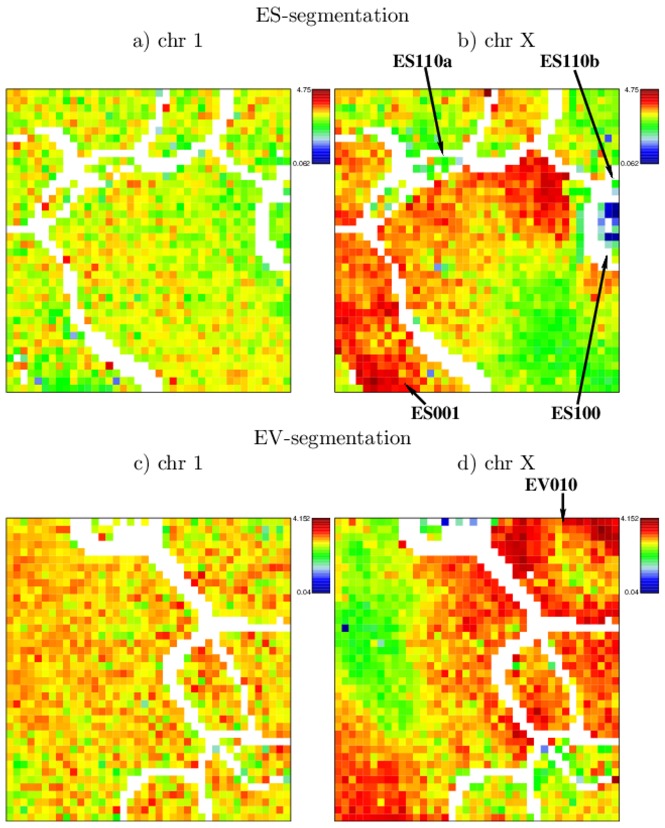
Chromosomal enrichment maps for chromosome 1 and X. The depict chromosomes show specific enrichment patterns. The logarithmic color scale ranges in a) and b) from 0.1 to 4.8 and in c) and d) from 0 to 4.2.

Any whole genome segmentation, per definition, provides a segmentation of the individual chromosomes. Thus, the results from the ES- and EV-segmentation can be displayed in individual SOM-images for each chromosome (see File S3 and S4). To emphasize differences in the assignment of segments from different chromosomes to the same node, we compute chromosomal enrichment maps. We therefore calculate the log ratio of the number of observed segments compared to the number of expected segments per node and chromosome (see Materials and Methods section for details). Data from most autosomes are similar and show an uniform distribution of chromosomal segments to subregions of the SOM, as depicted by Figures 8a and c for mouse chromosome 1. While SOM-images of chromosomes 12 and 14 are slightly speckled (see File S3 and S4), the areas showing up in the SOM-images of chromosome X correlate with the segmentation.

On chromosome X of a male individual, segments with the combinatorial epigenetic profiles ES100 and ES110 that are associated with poised or active chromatin in a broader sense are rare. On the other hand, segments with only H3K9me3 marks (ES001), associated with closed chromatin, are clearly overrepresented (see Figure 8b). Furthermore, segments marked with H3K4me3 in MEFs only (EV010) are particularly frequent on chromosome X with the exception of segments also associated with high amounts of H3K27me3 marks in ESCs (see Figure 8c). While a feature like the latter may be hard to explain with state of the art knowledge about epigenetic processes, it opens new questions on the genome-wide organization of chromatin states.

## Discussion

Analysis and, in particular, visualization of histone modifications on genome-wide scale is a difficult but promising task. Here, we present a novel SOM-based method for annotation-independent exploration of ChIP-seq data which combines genome segmentation, clustering and visualization of the histone modification patterns.

In the first step, our method requires the appropriate segmentation of the genome-wide data. The particular choice depends on the available data set as well as on the question under examination. Here, we make use of two different types of segmentations. One is based on different modification states in one selected cell type (ES-segmentation), the other based on one selected modification state in different cell types (EV-segmentation). While ES-segmentation mainly supports unraveling the epigenetic code relevant for genome organization and gene regulation, EV-segmentation can assist the formulation of hypotheses on the cross-talk between epigenetic states in the course of differentiation.

We would like to discuss briefly our method in comparison with other existing methods dealing with the segmentation of the genome based on ChIP-seq data. Ernst et al. introduced a segmentation method using Hidden Markov Models (HMMs) [35], which was recently published in the automated learning tool ChromHMM [36]. The position of annotated chromatin states greatly overlap the annotation of functional DNA elements such as enhancers and promoters. In ChromHMM, an epigenetic state is not described by a specific combination of histone marks (as in our method) but by the average enrichment of the marks. The higher dimensionality of the data used for segmentation enables a very detailed functional classification of chromatin states, however, also makes explorative analysis and comparative visualization of epigenetic patterns across multiple cell types much more difficult. Another segmentation tool is provided in the package ChromaSig, which was applied to nine chromatin marks for a part of the genome in human HeLa cells [37]. ChromaSig finds chromatin motifs of at least 2 kb length. A motif is not described by one value for each modification but by a statistical distribution of the reads over the motifs range. Specifically, ChromaSig allows to identify specific chromatin motifs for enhancer elements and promoter regions. Thus, both ChromHMM and ChromaSig offer a good view on functional aspects of epigenetics marks, but are restricted with respect to analysis of dynamic changes in modification patterns. Our method provides an alternative view, based on a combinatorial, annotation-independent segmentation of classes of different epigenetics marks and their correlated changes during differentiation. We have shown that one can draw conclusions about the underlying mechanisms and dynamics of epigenetics, e.g. about global switching events in chromatin states during differentiation.

Different methods to cluster and visualize high-dimensional data have been established; among them principal component analysis, hierarchical clustering, non-negative matrix factorization and visualization techniques such as heatmaps, network-representations and dendrograms with particular advantages and disadvantages [38]. One of the neuronal network-based methods is the SOM-method. We applied SOMs because it combines clustering, multidimensional scaling and visualization in one method. Moreover, the SOM-method provides an intuitive and global view on patterns of epigenetic marks allowing straightforward exploration of biological hypotheses. Our examples demonstrate that visual inspection of the obtained SOM-atlas provides detailed insights into genome-wide reorganization of the epigenome in the course of cellular differentiation such as *de novo* formation of poised and active chromatin.

A further advantage of SOMs is the simple way to integrate additional information, e.g., on the lengths of the segments carrying modified histones or on specific sequence motifs associated with these modifications such as the CpG-density. This combined information is important for the evaluation of hypothesis on the particular mechanism of recruiting histone marks. For example, our SOM-analysis suggests CpG-independent modes of stable maintenance of H3K4me3. Moreover, recruitment of histone marks to short and CpG-poor segments during development of ESCs must be driven by a CpG-independent, likely sequence specific, binding of the respective modifying complexes. SOM-analysis allows identification and selection of genomic regions potentially containing such so far unknown binding motifs. We predict that segments of the EV111-type contain motifs enabling the recruitment of methylases to H3K4. In addition, information about gene expression can be directly integrated into SOM-analysis. Association of the segments with the expression levels of overlapping genes enables studying epigenetic regulation of transcription. As an example, we here identified CEPs associated with housekeeping genes, e.g. ES100.

Exhaustive human epigenetic data sets are currently in preparation in large scale projects like the Roadmap Epigenomics Project [39] and the Human Epigenome Project [40]. They comprise epigenetic and genetic information for various cell types and for individuals of different age. The resulting combinatorial explosion for possible realizations of, e.g., different MSs and different combinations of supporting data poses particular challenges for SOM-analysis. In particular, the resulting SOMs may become too complex for a straight-forward analysis by direct visual inspection. Consequently, a comparison of epigenetic patterns between different tissues and/or different individuals requires a method for automated comparison of SOM-images. Such a systematic visualization of similarities between individual SOM-images enables a powerful second level analysis of the data sets. In the field of gene expression analysis, the resulting patterns are known as “metagene expression patterns” and have been applied to generate differentiation-related catalogs of global gene expression states [25]. Thus, a combination of our method with this type of high-level analysis is well suited to generate a catalog of epigenetic modification patterns for different cell types of different ages.

Aside from histone modifications, other types of signals can be measured genome-wide using high-throughput sequencing, e.g. transcription factor binding to DNA or the whole transcriptome. Comparing such signals among each other and selected cell types may reveal new relations between the underlying processes. Such data sets can be explored with our method to provide a first, annotation-independent but genome-wide view at the data. Hence, our method is not restricted to the exploration and analysis of correlations in chromatin modifications, but can be applied to a much wider range of genomic data. Thereby, it helps to guide studies into promising directions.

### Conclusion

We presented an approach for segmentation of the genome with respect to either different epigenetic marks in one cell type (ES-segmentation) or one epigenetic mark in different cell types (EV-Segmentation). The approach allows lossless compression of the information about epigenetic states and is followed by sorting, clustering and visualization via self-organizing maps. Furthermore, complementary quantitative features obtained from the genomic sequence, localization or gene expression can be explored to detect possible correlations with the modification states. The method is unique as it actually provides a global view of genome-wide epigenetic information. Already in its simplest form, meaning without integration of additional information, the exploration tool allows tracing formation and disappearance of combinations of different histone modifications over the considered cell samples. Systematic investigation of correlations between epigenetic states and genomic or organizational features are likely to reveal novel modes of epigenetic regulation and will thereby shed light on epigenome-associated information processing in living cells.

Our method provides a versatile interface to analyze and present data sets in a way that strongly facilitates formulation of new hypotheses. Besides generating intuitive visualizations of genome-wide dependencies, the method conserves the full information available in the original data sets. It allows comprehensive downstream analyses in terms of alternative approaches and appropriate statistical testing. These issues are beyond the scope of this work.

## Materials and Methods

### Details on the SOM-algorithm

We applied SOMs with 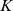 nodes arranged on a 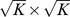 square grid. The algorithm starts with a linear initialization of the meta-EPs assigned to SOM-nodes. Linear initialization extracts the two major properties (projections) structuring the data using principle component analysis. The first two principle components are mapped onto the width and height of the SOM, respectively. After initialization of the SOM the algorithm continues with a learning procedure iterated over all EPs in a sequential manner. In each of the iterations 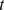, the EP of a given segment is assigned to the most similar meta-EP in the SOM according to Euclidean distance in the 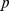-dimensional EP-space. In addition, all 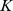 meta-EPs are adjusted as well in each iteration using the following learning rule: Let 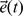 be an input EP with its most similar meta-EP in SOM-node 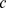. Then for each meta-EP 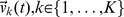 the updated meta-EP 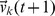 is calculated by
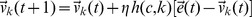
with adaptation gain 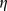 (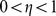) and neighborhood function 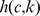. A full number of iterations over all EPs is called *epoch*. The adaption gain 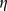 is decreased steadily in a linear manner over subsequent epochs [27]. The neighborhood of a node 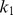 is defined according to a two-dimensional normal distribution around 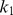. This so called Gaussian neighborhood is maximal for nodes adjacent to 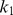 and decreases asymptotically to zero for nodes located distantly in the SOM-grid [41]. For any two nodes 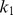 and 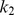, the neighborhood function is defined as
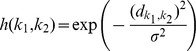
where 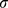 denotes the neighborhood radius and 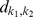 the Euclidean distance of 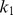 and 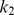 on the SOM-grid. The neighborhood radius 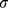 is steadily decreased over subsequent epochs.

### Construction of Supporting Maps

We prepared data sets on a) the average length of segments mapped to a particular node, b) the average CpG-density of mapped segments, c) the fraction of segments that exclusively overlap with genes which are significantly expressed in all three cell types (present-call map) and d) the fraction of mapped segments overlapping with developmental genes and the data set on e) the fraction of mapped segments associated with a specific chromosome. The data are computed and integrated into SOMs in the following ways:

- segment length map: The length of a segment is given by the number of nucleotides in the segment. Due to the procedure described in subsection Genome Segmentation, we obtain segment lengths of 200 nt or larger. SOM-nodes are colored according to the average length of segments assigned to the node.
- CpG-density map: We define the CpG-density of a segment as the number of CG-dinucleotides within the segment's DNA sequence divided by the length of the segment. For the calculation, we used the DNA sequence obtained from the plus strand as defined in the mm9 genome. The node value, finally represented by the color of the tile, is the average over the CpG-density of all segments assigned to a node.
- present-call map: In order to calculate the expression status of a segment, we first mapped the transcripts from the gene expression chip, published in [11], to the segments. Therefore, we used the ensemble data base and mouse genome mm9. Since histone modifications at promoter and enhancer sequences upstream of the transcription start site play a major role in transcriptional regulation, we added 2000 nt to the 5′-end of each transcript represented by the chip. The expression data sets are preprocessed as described in [42]. In this preprocessing step, the genes are classified into present/expressed or absent/not expressed. We assigned an expression value of 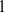 to expressed genes and 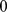 otherwise. For each segment, we averaged over all assigned expression values. Only segments with an average expression value of 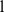 are considered as “active”. In the present call map, SOM-nodes are colored according to the fraction of segments considered as active.
- developmental genes map: We downloaded the GO-Term annotation for all genes in the ensemble data base and selected all genes assigned to the term “cellular developmental process” to create a list of developmental genes. Using the assignment of genes to segments from c), we count for each node the number of developmental genes assigned to the node and normalize this number by the total number of assigned genes.
- chromosome-specific population maps and chromosomal enrichment maps: For each node 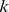, we count the total number of segments 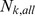 and the number of segments 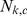 originating from chromosome 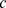. Furthermore we calculate the total number of segments originating from chromosome 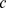, 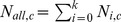 and the total number of segments 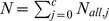. The expected number 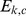 of segments from chromosome 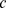 assigned to node 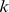 is therefore the expectation value of a hyper-geometric distribution. It is given by 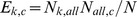. The fraction of segments originating from chromosome 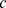, i.e. 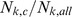 is the node value of node 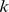 in the chromosome-specific population map, while 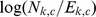 is the node value of node 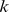 in the chromosomal enrichment maps for chromosome 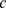.

The default color scale of the derived heat maps ranges from blue over green to red. While low values map to cold colors (blue), high values map to warm colors (red). Due to the insufficient discrimination of color intensities in the blue range, we inverted the color scale leading to a better perception of smaller values in the length map.

## Supporting Information

File S1 **Varying the relative contribution of the coverage part of the EP.** The ES-segmentation SOMs are recalculated with several weights for the coverage part of the EPs to show the impact on the resulting SOM structure.
(PDF)

File S2 **Parameter validation for the SOM.** The arbitrary parameters for the training procedure, i.e. width and height of the SOM, are altered to determine the correct size of SOM. Resulting SOM-atlases for both segmentations and different parameter values are shown.
(PDF)

File S3 **Additional SOM images for ES-segmentation.** Chromosomal-specific population map and chromosomal enrichment maps for ES-segmentation are shown for all chromosome in the mouse genome.
(PDF)

File S4 **Additional SOM images for EV-segmentation.** Chromosomal-specific population map and chromosomal enrichment maps for EV-segmentation are shown for all chromosome in the mouse genome.
(PDF)
