# Supplementary material for: A Global Genome Segmentation Method for Exploration of Epigenetic Patterns
Source: PLoS One. 2012 Oct 12;7(10):e46811. doi: 10.1371/journal.pone.0046811 (PMC3470578; doi:10.1371/journal.pone.0046811)
Supplement: File S1 — Varying the relative contribution of the coverage part of the EP. The ES-segmentation SOMs are recalculated with several weights for the coverage part of the EPs to show the impact on the resulting SOM structure. (PDF) [file pone.0046811.s001.pdf]

Varying the relative contribution of the coverage part of the EP

The epigenetic profile (EP) represents a vector of length $p = n \times m$ (n - number of histone modifications; m - number of cell types) which divides into CEP- and coverage (COV)- subvectors of length n and $n \times (m-1)$ upon ES-segmentation, respectively. Upon EV-segmentation their lengths are m and $m \times (n-1)$, respectively. The components of the coverage subvector are weighted by a factor w so that the EP can be formally written as EP=(CEP, $w*$COV).

To assess the impact of the weighting factor on the resulting SOM structure we varied its value between $w = 1/10$ and 10 and trained new SOMs (see Figure below). The population maps reveal that low and moderate coverage weights ($w \leq 2$) ensure SOM structures with well pronounced CEP-islands which are separated by low-populated border lines (see blue regions in the population maps in the figure). With increasing $w > 2$ these border lines tend to smear because adjacent CEP-islands progressively overlap. On the other hand, with decreasing $w < 2$ the CEP structure becomes more clearly expressed and split CEP islands tend to merge, e.g. for CEP=(010) and (001) (compare the population maps for $w = 2$ and $w = 0.1$).

Hence, at small $w \leq 1$ the SOM images are dominated by the different CEPs where the respective CEP-islands modify in the different states in terms of newly arising or disappearing red and blue areas due to de-novo methylation or de-methylation of the respective histone residues, respectively. Recall that our segmentation approach relates the three different histone modifications in MEF and NPC to a reference defined by the respective CEP in ESC (for ES-segmentation) or, alternatively, the H3K27me3 and H3K9me3 modifications to the H3K4me3 reference CEPs in three different cell systems (for EV-segmentation). The changes of the modification patterns can be directly related to the reference states given by the respective CEPs for low and intermediate values of w.

At larger value $w > 2$ the low-populated borders between adjacent CEP-islands progressively populate with EPs and finally disappear due to the growing weight of the continuous coverage values. In consequence the SOM-images become smoother showing a reduced number of spots where red and blue areas refer to high and low modification degrees of the coverage components of the EP-components, respectively. Hence, at larger values of the weighting factor the relation to the reference state partly disappears. On the other hand, concerted changes of the modification states as expressed by the respective coverage components of the EPs tend forming unique spots which allows identifying groups of correlated features independent of their initial CEP. Such correlated sets of segments are candidates for functionally related regions of the DNA. Hence, SOM patterns dominated by the coverage part of the EPs are suited for extracting common regulatory modes in the data.

We here focus on the former issue namely to study changes related to the reference state. In the system studied the weighting factor $w = 1$ provided

good results in terms of SOMs which are well structured according to the CEP. However, it might be advisable to use $w < 1$ in future applications with a larger number of coverage components of the EP (e.g. if one considers a larger number of histone modifications and/or of cell systems) to ensure that the basal structure of the SOM is determined by the underlying CEP states. In such applications we suggest $w \sim 2/(m-1)$ and $w \sim 2/(n-1)$ for ES- and EV-segmentations, respectively.

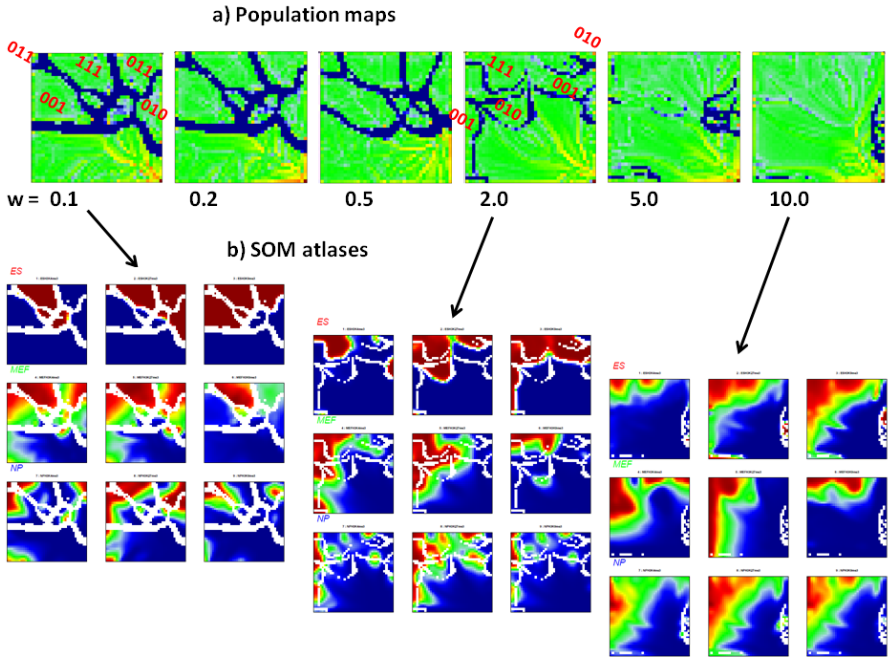

Figure 1: SOM trained with varying weights $w = 0.1...10$ of the coverage components of the EP. Panel a shows the population maps (blue-green-red refers to low-intermediate-high numbers of segments per tile according to the scale defined in the main paper) where selected islands were annotated according to their CEP. Note for example, that the CEP=(001) and (010) forming single islands at small values $w \leq 2$ split into two sub-islands at $w = 2$. Panel b) shows the SOM atlases for selected $w = 0.1, 2$ and 10 after ES-segmentation. The color code is the same as used in the main paper.
