# Supplementary material for: A Global Genome Segmentation Method for Exploration of Epigenetic Patterns
Source: PLoS One. 2012 Oct 12;7(10):e46811. doi: 10.1371/journal.pone.0046811 (PMC3470578; doi:10.1371/journal.pone.0046811)

Parameter validation for the SOM

We run the SOM training procedure also for different sizes. The results for 20×20, 30×30, 50×50, and 60×60 are shown for both segmentations below. For SOM sizes smaller than 40×40 square grid, we see that islands are not separated completely or can not be found at all in the SOM image. Larger sizes of the square grid result in empty nodes meaning that there are not enough different epigenetic profiles for filling the amount of nodes in the SOM. Thus, the results on the 40×40 grid are used for interpreting the data. In the caption of each figure the disadvantages of the SOM iamge size are explained.

ES-segmentation

Figure 1: SOM gallery for size 20 × 20: Islands ES110a, ES010a, ES110b and ES101 can not be found. The border between islands ES001 and ES111 can only hardly be detected. Thus, we can not see all CEP and would miss associated effects.

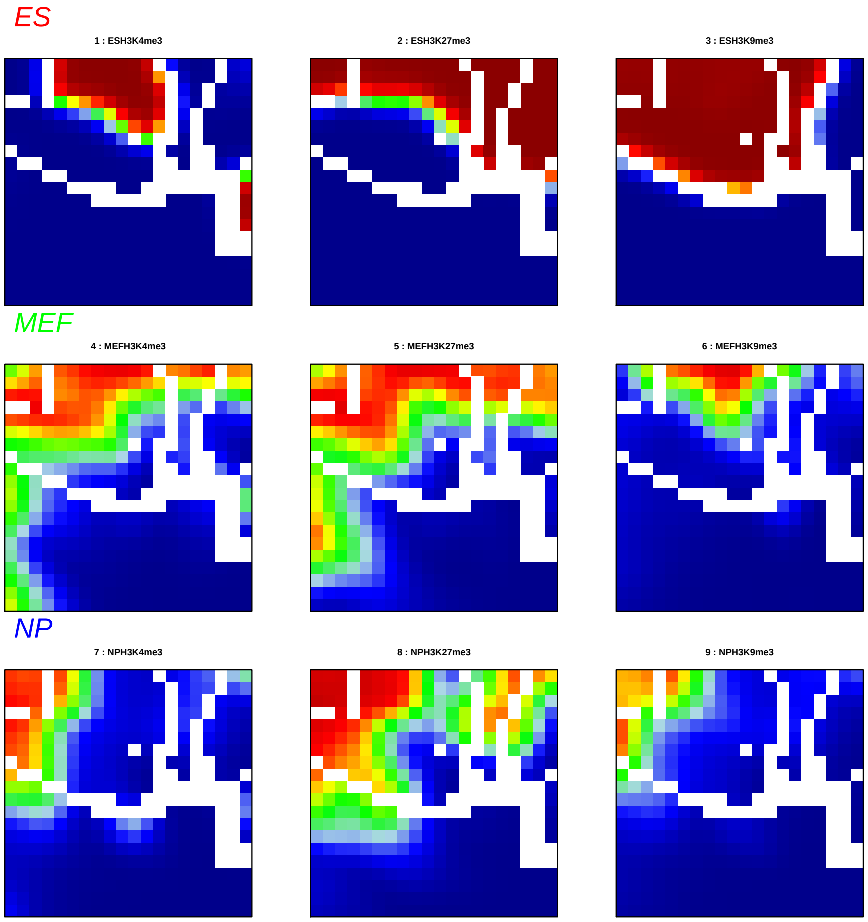

Figure 2: SOM gallery for size 30 × 30: Island ES101 is missing. Effects associated with ES101 can not be detected. ES010a and ES110a as well as ES110b and ES100 are not isolated clearly making it hard to decide whether the manifestation of features is restricted to one of islands in such a pair or not.

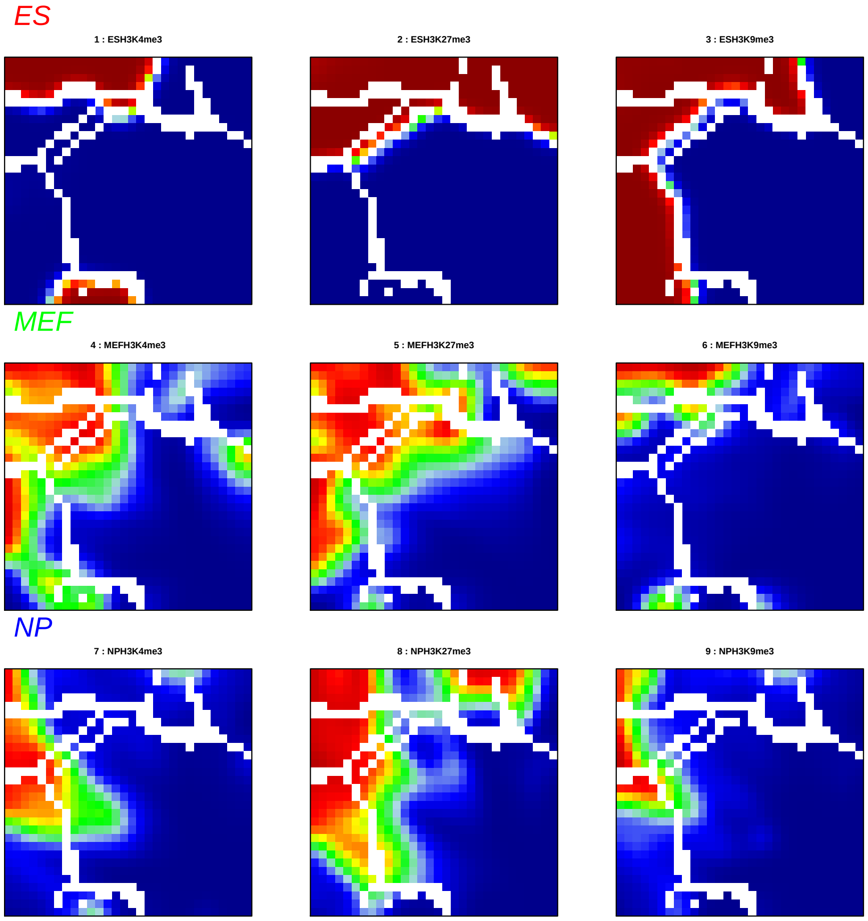

Figure 3: SOM gallery for size 50 $\times$ 50: In ES100 and ES000 one can found the first segment-free nodes that do not belong to borderlines. Especially, the segment-free nodes in ES000 look like borderlines. However, the separated node shows the same modification pattern as ES000 and thus is part of ES000.

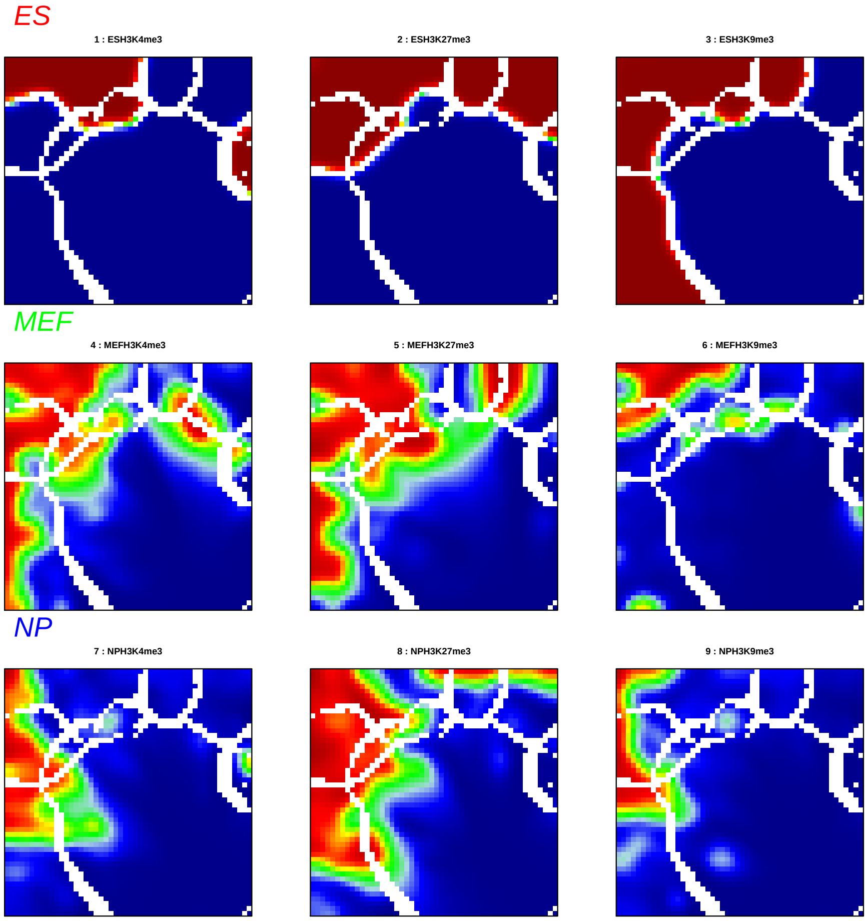

Figure 4: SOM gallery for size 60×60: In ES010b and ES000 empty nodes are located which do not belong to borderlines. The separation of the node in ES000 by segment-free nodes is even stronger than in the 50×50 SOM images. The separation of this node would lead to false interpretation of the results.

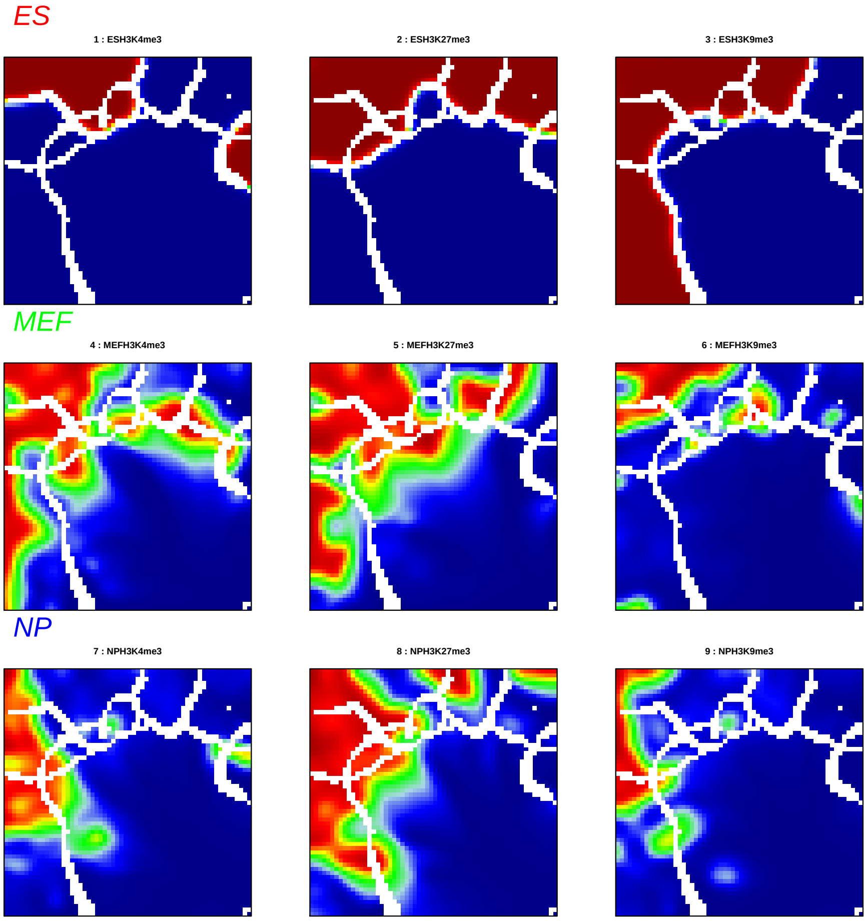

EV-segmentation

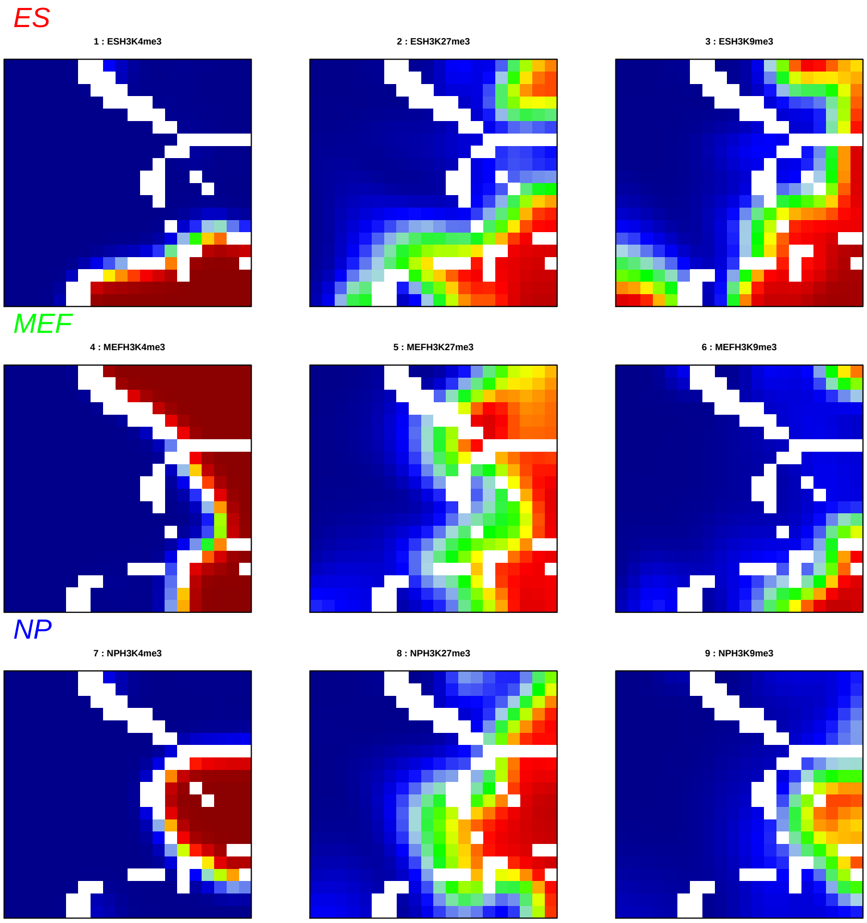

Figure 5: SOM gallery for size 20 × 20: Island EV100b and EV101 are missing. Specific characteristics of this islands will be missed. EV111, EV110, and EV100a form one island and can not be distinguished. The borderline between EV001 and EV011 is not very pronounced. Associating the manifestation of a feature with these islands is hard.

Figure 6: SOM gallery for size 30×30: Island EV101 is missing. The borderline between EV011 and EV001 is incomplete. This makes it difficult to decide whether the manifestation of features is restricted to one of the islands in such a pair or not.

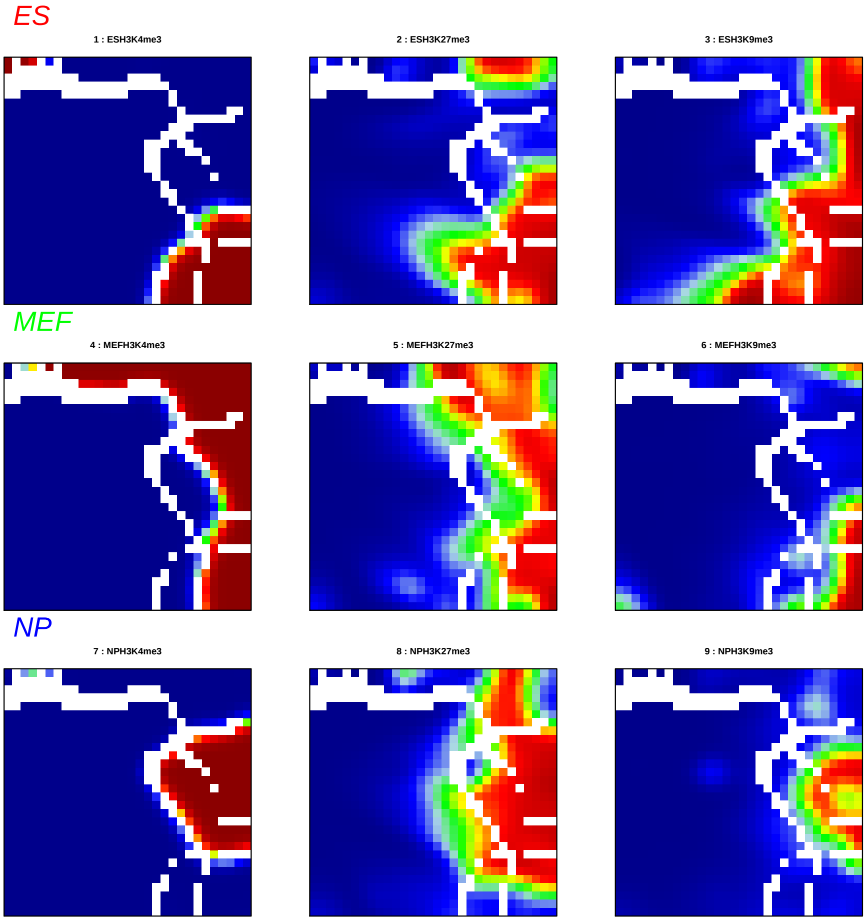

Figure 7: SOM gallery for size 50 × 50: There are empty nodes in EV000 and EV100b. The unpopulated node located in islands EV100b may be interpreted as being an incomplete borderline which leads to wrong implications.

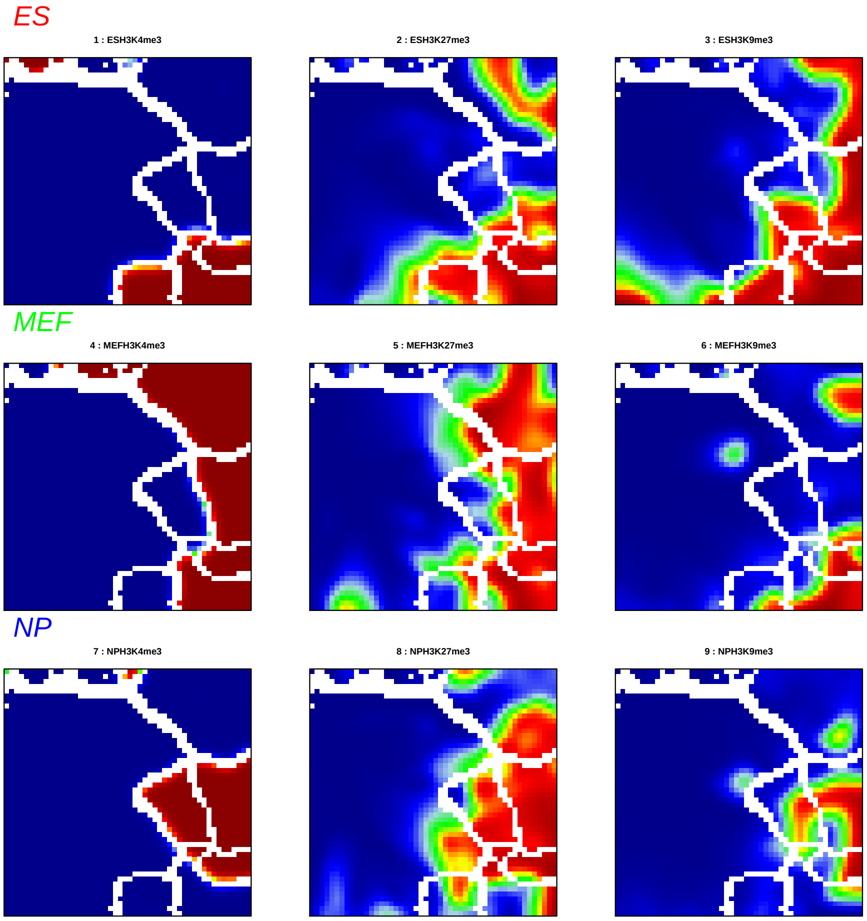

Figure 8: SOM gallery for size 60 × 60: There are empty nodes within several islands. One may assume that they belong to borderlines. This leads to wrong implications.

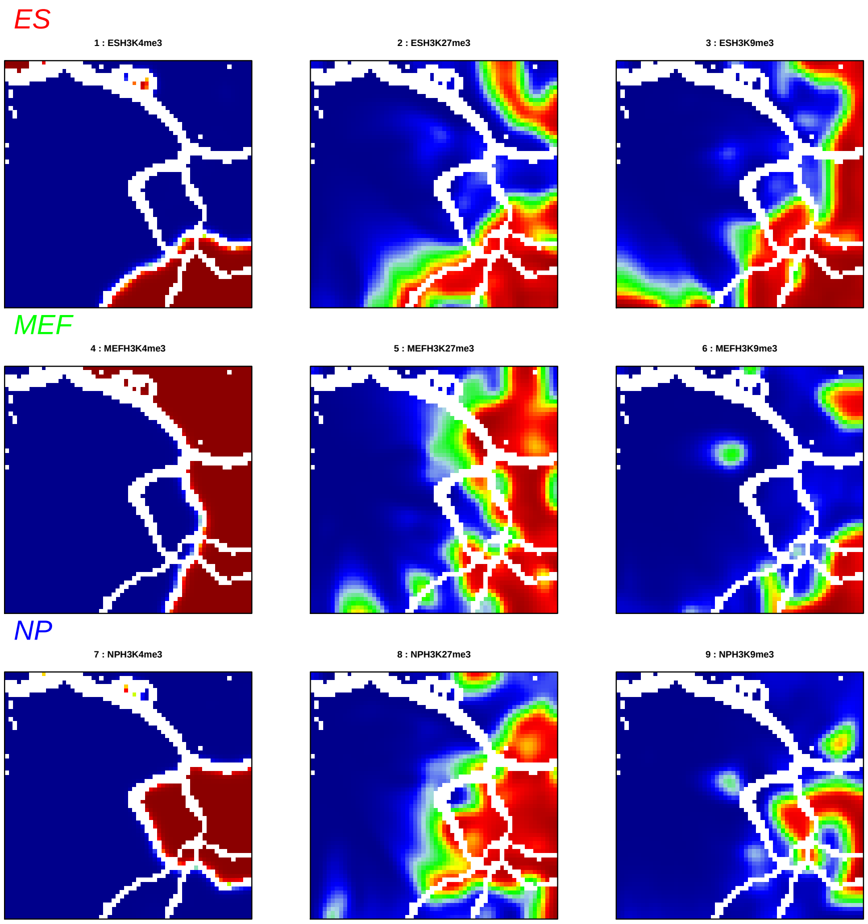

Supplement: File S2 — Parameter validation for the SOM. The arbitrary parameters for the training procedure, i.e. width and height of the SOM, are altered to determine the correct size of SOM. Resulting SOM-atlases for both segmentations and different parameter values are shown. (PDF) [file pone.0046811.s002.pdf]
