# Supplementary material for: A Global Genome Segmentation Method for Exploration of Epigenetic Patterns
Source: PLoS One. 2012 Oct 12;7(10):e46811. doi: 10.1371/journal.pone.0046811 (PMC3470578; doi:10.1371/journal.pone.0046811)
Supplement: File S4 — Additional SOM images for EV-segmentation. Chromosomal-specific population map and chromosomal enrichment maps for EV-segmentation are shown for all chromosome in the mouse genome. (PDF) [file pone.0046811.s004.pdf]

Additional SOM images for EV-segmentation

Chromosomal-specific population maps

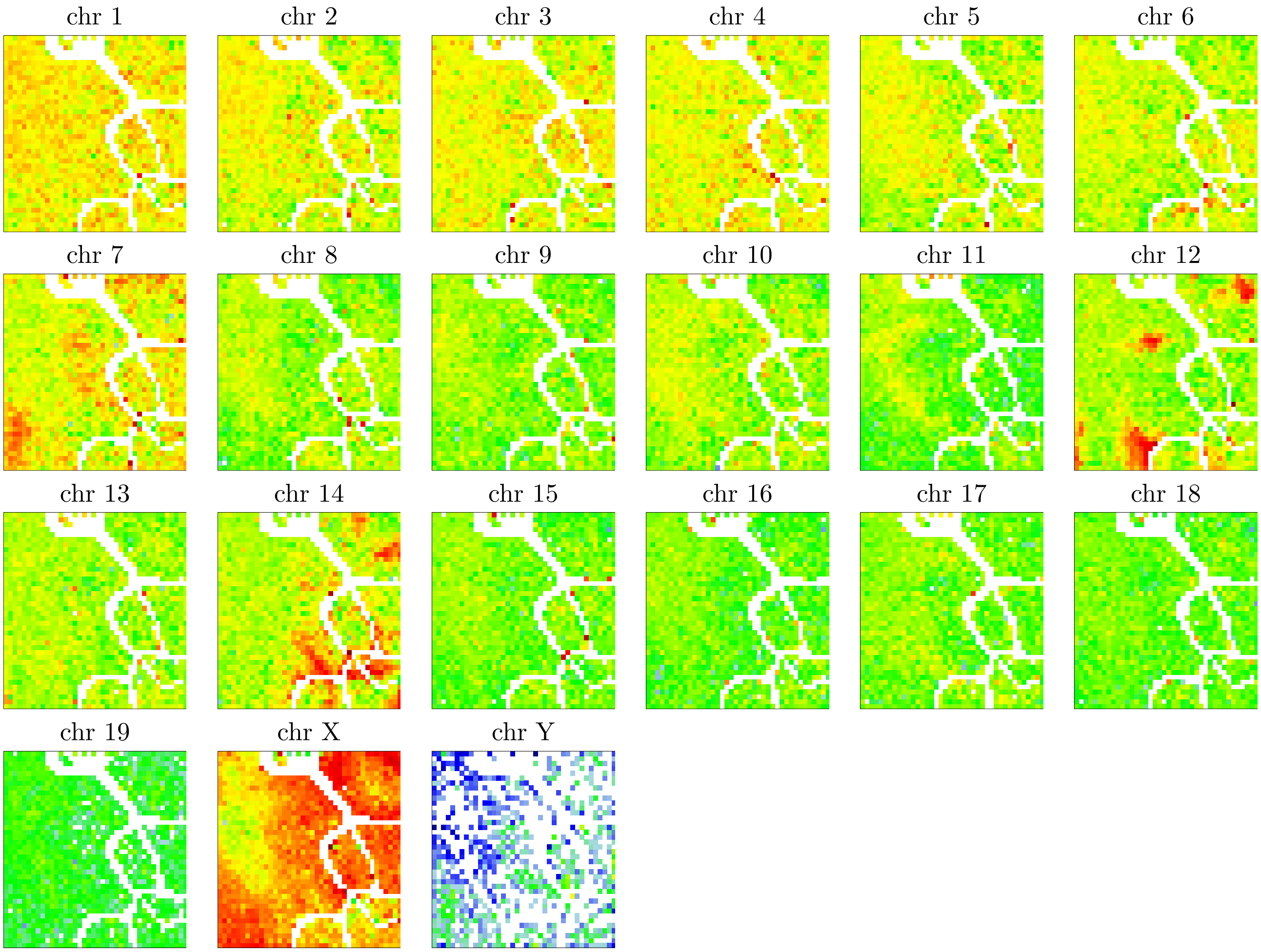

Figure 1: Chromosomal-specific population map of EV-segmentation for each chromosome. The logarithmic color scale ranges from 0 to 1.

Chromosome enrichment maps

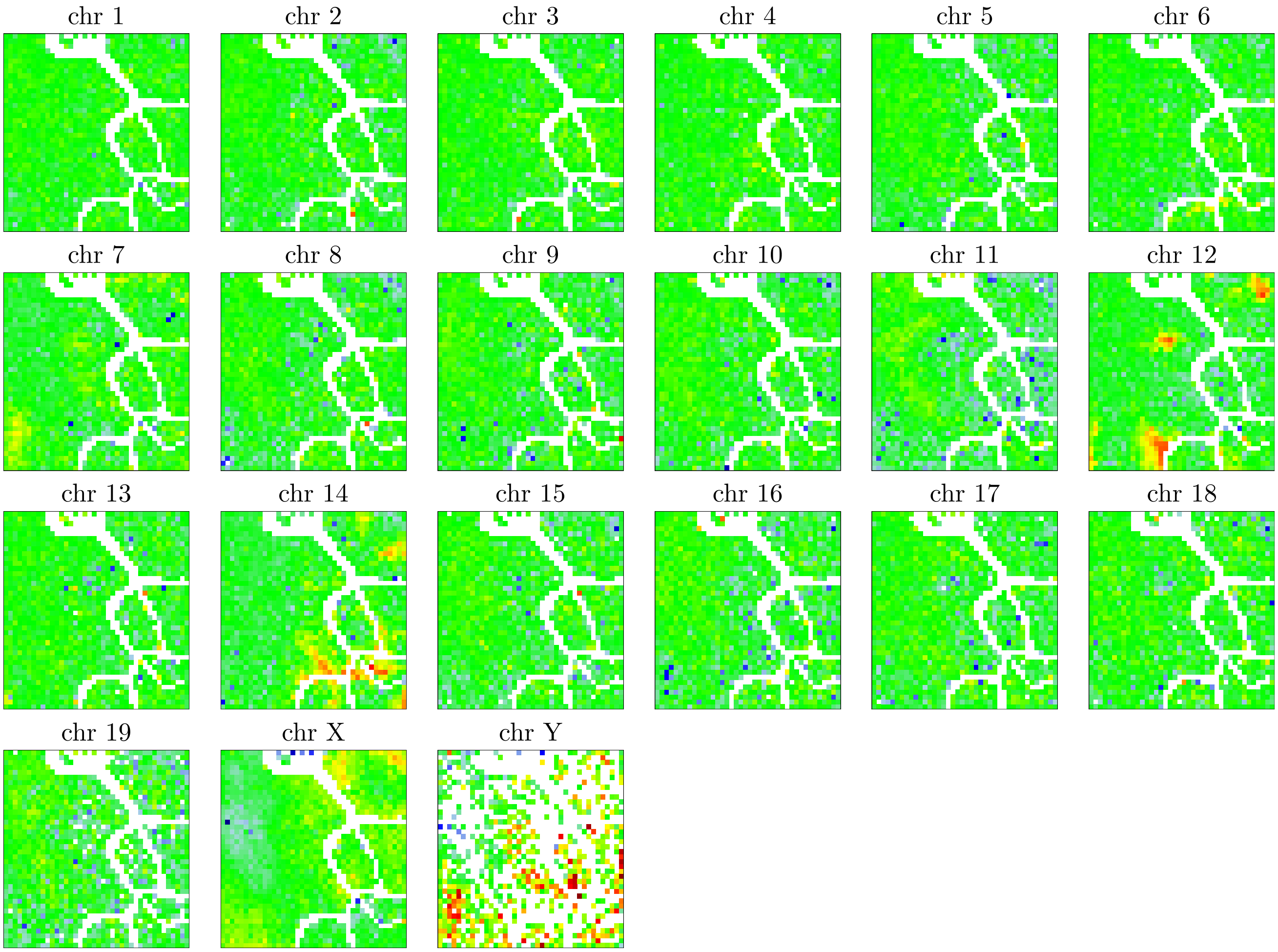

Figure 2: Chromosome enrichment maps of EV-segmentation for each chromosome. The logarithmic color scale ranges from 0 to 24. Light blue corresponds to the expected number of segments within one node (enrichment of 1). Nodes with a 10-fold enrichment are colored in orange.
